# Factors Influencing Consumers’ Willingness-to-Try Seafood Byproducts

**DOI:** 10.3390/foods12061313

**Published:** 2023-03-20

**Authors:** Silvia Murillo, Ryan Ardoin, Witoon Prinyawiwatkul

**Affiliations:** 1 Agricultural Center, School of Nutrition and Food Sciences, Louisiana State University, Baton Rouge, LA 70803, USA; smurillomiguez1@lsu.edu; 2 Food Processing and Sensory Quality Research Unit, Southern Regional Research Center, USDA-ARS, New Orleans, LA 70124, USA; ryan.ardoin@usda.gov

**Keywords:** byproduct, seafood, willingness to try, product appropriateness, emotions, safety claim, health benefit claim

## Abstract

With increasing global demand for seafood, seafood byproducts (SB) utilization can contribute to a more sustainable food supply chain through waste-to-value food product development. However, consumer perceptions of SB (e.g., fish skin and bones) are underexplored. Therefore, this study aims to evaluate some factors influencing consumers’ willingness-to-try seafood byproducts. An online survey was conducted in the USA regarding intervention of SB informational cues with N = 904 adult seafood consumers internationally. The proportion of consumers willing to try SB increased significantly (McNemar’s test, α = 0.05) from 47% to 68% after SB safety and health claims had been presented in the questionnaire. Gender, race, SB knowledge, and previous SB consumption were significant predictors of trial intent (based on logistic regression), as were emotional baseline scores during the COVID-19 pandemic. Males were more open to SB consumption than females, and racial identity was associated with differential responsiveness to SB information. Higher levels of “bored” and “unsafe” feelings, and lower levels of “free” were associated with increased SB trial intent. Potential SB consumers identified fish products (82% willingness-to-try); seasoning mix, sauces, and dressing (71% willingness-to-try); and soup and gravy products (62% willingness-to-try) as most appropriate for SB incorporation. Predominant reasons for SB avoidance were concerns about sensory quality, safety, and nutrition. These consumer-driven data could guide SB product development concepts to encourage trial and overcome aversions through new consumption experience.

## 1. Introduction

Reducing food loss and waste has been among the most significant challenges facing sustainable food systems and global food security [1]. It has been estimated that almost one-third of food produced for human consumption is wasted every year, and the amount of food loss is expected to increase with population growth and consumption [2]. Meat, poultry, and fish have accounted for approximately 30% of total food value lost [2], with the global demand for seafood increasing faster than that of terrestrial meat [3]. Global per capita seafood consumption has grown by a rate of 122%, from 0.9 kg in 1961 to 20.5 kg in 2019 [4]. Generation of seafood loss, byproducts, and waste can occur throughout the supply chain, from harvesting to processing, distribution, and consumption [5]. During fish processing, for example, almost 55% of biomass can be diverted to non-food production streams [2] such as cosmetics, pharmaceuticals, fishmeal, and animal feed, or be discarded. This includes potentially edible byproducts with nutritional value [6]. The entire seafood industry generates about 60% of byproducts which are mainly skin, viscera, bones, fins, belly flaps, heads, exoskeletons, and blood. For example, fish filleting generates 200 to 400 kg of byproducts per ton. The crustacean industry follows the same fashion as the fish industry, generating around 80% of byproducts of the total weight of prawns, shrimps, crabs, and lobsters [7,8].

In the hierarchy of biomass value, pharmaceutical applications yield a higher economic value than human food applications [9]. However, societal value of keeping valuable nutrients in the food supply chain should also be considered. With current and projected rates of seafood production (and subsequent byproduct generation) and the variety of SB sources and composition [10], food applications of SB could work alongside other production streams to reduce waste rather than be in the competition. Strategies to progress toward more sustainable food systems have included related concepts of circular economy, byproduct valorization, and waste-to-value production [11,12]. These approaches aim to minimize food loss by keeping edible components of dietary or nutritional value in the food production system. This often involves new food applications of ingredients which may be unfamiliar to consumers [11,12]. Commercialization of these products needs in-depth research on consumers to overcome barriers such as consumer perception, unfamiliarity, negative emotions, and potential risk that can decline the willingness-to-try a new product.

Prior research found that appropriate product concepts could encourage consumers’ willingness-to-try novel food sources and emphasized that appropriateness of an ingredient within a product is best determined by the potential users/consumers [13]. The willingness-to-try response is a measure of consumers’ intent or “willingness” to engage in a hypothetical behavior (for instance, seafood byproduct consumption). Such metrics are common in survey-based studies of novel foods, where actual behaviors cannot be observed, and/or past opportunities may not have been presented [14]. The most common drivers of consumers’ willingness-to-try novel food products are positive emotions, sensory attributes, product appropriateness, familiarity (previous positive experiences), knowledge (product information), and education. In contrast, some factors that inhibit consumers’ willingness-to-try products include unfamiliarity with the products or raw materials, cultural preferences, negative emotions, and food neophobia [13,14,15,16]. Some food applications of SB have shown promise with consumer segments (e.g., catfish bone powder in a seafood breading mixture [17]), others have demonstrated disconfirmed sensory expectancy on the perceived product performance (e.g., dark orange color in dipping sauces containing shrimp head pigment [18]). With global demand for the production of seafood rising [3], there is increasing need for consumer-driven product development concepts to keep valuable seafood components in the human food supply.

Some consumers have demonstrated negative psychological reactions to consumption of byproducts, despite potential benefits of sustainable food sources [11]. Barriers to adoption of foods containing unusual ingredients such as insects [13] and animal byproducts [15] have been noted, but consumers’ openness to SB consumption has been underexplored on a large scale. Along with appropriate products, communication of ingredient safety and potential benefits have been suggested to overcome negative attitudes and emotions which may hinder trial of unfamiliar foods derived from byproducts [11,15]. This current study will provide essential data such as appropriateness, perceived risk, that can be applied by product developers to create novel products. The objectives of this research were to (1) evaluate consumers’ openness to SB consumption under different [increasingly] informed conditions (definition only, safety informational cue, and health benefit informational cue), (2) reveal both emotional and demographic predictors of SB consumption, (3) determine appropriate food product categories for SB application, and (4) identify potential risks associated with the unwillingness to try SB.

## 2. Materials and Methods

### 2.1. Online Survey and Data Collection

**Figure 1:**
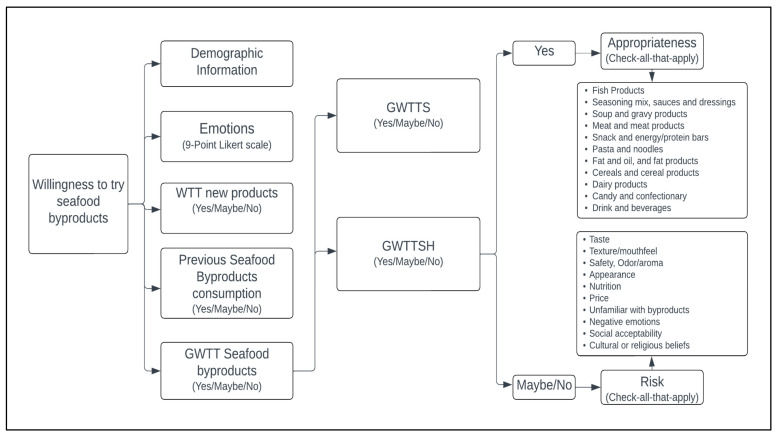
Schematic overview of the online survey work. WTT: Willingness-to-try, GWWT: General willingness-to-try [with seafood byproduct definition only], GWTTS: General willingness-to-try [with definition and safety information], GWTTSH: General willingness-to-try [with definition, safety information, and health benefit information].

An online survey was designed and administered using Qualtrics online software (Qualtrics Provo, UT, USA). Weblinks and QR Codes were generated to distribute the survey through email, online platforms, social media, and a consumer database (“Tiger Tasters”, Baton Rouge, LA, USA). Potential respondents were screened for age (at least 18 years old) and seafood consumption (“Do you consume seafood or fish products?” = yes). Participation was entirely voluntary, and informed consent was collected. The questionnaire and research protocol were approved by Louisiana State University Agricultural Center Institutional Review Board (Baton Rouge, LA, USA; IRBAG-21-0063). Data were collected online from April to June of 2020, the months immediately after COVID-19 was declared a global pandemic [19]. Figure 1 exhibits the schematic overview of the survey, which was completed by a total of 904 adult seafood consumers. To achieve the study goal, the survey was designed to allow each step to collect essential data to predict the willingness-to-try (GWWT: General willingness-to-try [with seafood byproduct definition only]; GWTTS: General willingness-to-try [with definition and safety information]; GWTTSH: General willingness-to-try [with definition, safety information, and health benefit information]) based on demographics, emotions, prior consumption experience, and informational cues, to identify the to p products appropriate for incorporation of seafood byproducts, and to identify factors (risks) inhibiting consumers’ willingness-to-try seafood byproducts. Details for the methods in each step are given below.

### 2.2. Demographic Information

Over half (62%) of the respondents were considered US consumers (“Have you lived in US for the last three years?” = yes) and non-US consumers were not asked to report their nationality, so their locations were unknown. Sixty-three percent of respondents were female. Proportions of the population sample by race were as follows: 39% Caucasian, 37% Hispanic/Latin/Spanish origin, 19% Asian, 3% Black/African American, and 2% other races. The largest age segment was 26- to 35-years-olds (37%), followed by ages 19–25 (17%), 36–45 (16%), 46–55 (15%), 56–65 (9%), and 65 or older (6%). The majority of the respondents (94%) were college-educated, and about 76% were employed either full-time or part-time at the time this survey was administered (Table 1).

Eighty-three percent of consumers responded “yes” to the question “Are you typically willing to try new foods?” An additional 14% responded “not sure.” While this was not a measure of food neophobia per se, it indicated that most respondents were at least open to the idea of new food experiences. Seventy-six percent of consumers claimed to know what seafood byproducts were. Regardless of their responses, all participants were subsequently presented with the following SB definition: “Related to seafood and aquacultural products, a byproduct is something (for example, bone, skin, gut) which is produced during the manufacturing or processing of another product (for example, catfish fillet).” After presentation of this SB definition, 46% of participants responded “not sure” to the question, “Have you ever eaten food products that contain or are fortified with seafood or aquacultural (farm-raised) byproducts such as head, bones, gut, or skin?”; 39% responded “yes.” 

### 2.3. Emotional Profiles

Consumers rated 21 baseline emotions in response to the question “How have you been feeling during the COVID-19 pandemic?” Twenty-one emotion terms (selected from the EsSense Profile^®^ [20]) were presented in a randomized order and rated using a bipolar 9-point Likert scale (disagree extremely, disagree very much, disagree moderately, disagree slightly, neither disagree or agree, agree slightly, agree moderately, agree very much, agree extremely) with a tenth “not applicable” option. The list included the following emotions: active, adventurous, aggressive, bored, calm, eager, energetic, enthusiastic, free, friendly, good, glad, happy, healthy, loving, nostalgic, pleased, peaceful, satisfied, unsafe, and worried. Baseline emotional profiles obtained from the data were treated as psychographic predictors of SB consumption.

### 2.4. Willingness-to-Try Seafood Byproduct, Perceived Risks, and Product Appropriateness

In this experiment, consumers’ general willingness-to-try foods made with SB was measured under three informed conditions. Participants were first asked, “Would you be willing to try new products containing a small portion of seafood byproducts such as bone and skin?” (yes/maybe/no). This response was subsequently referred to as the general willingness-to-try (GWTT) response and was obtained given only the SB definition provided earlier. Next, participants answered the question with an additional safety informational cue: “Would you be willing to try new products containing a small portion of seafood byproducts such as bone and skin, knowing it is safe for consumption?” (yes/maybe/no). This willingness-to-try response under the informed condition of SB safety was later referred to as (GWTTS). Finally, consumers answered the question, “Would you be willing to try new products fortified with safe seafood byproducts that are claimed to provide health benefits?” (yes/maybe/no). This response, with an added informational cue related to potential SB health benefits, was referred to as GWTTSH.

Consumers who responded “maybe” or “no” to the GWTTSH question were considered reluctant or averse consumers, respectively, and directed to answer the question “Why?” and reported the reasons for their response from a list of perceived risks [13,20] in a check-all-that-apply (CATA) format. The list of risks included: taste, appearance, odor/aroma, texture/mouthfeel, safety, nutrition, negative emotions (boredom, disgust, fear, guilty, worry, etc.,), social acceptability, cultural or religious beliefs, unfamiliar with byproducts, and price. This list was meant to identify potential negative consequences consumers may anticipate from SB consumption (and thus avoid it), and to encompass the four dimensions of risk perception presented by Baker et al. [16]: functional, physical, social, and psychological risks.

Consumers who responded “yes” to the GWTTSH question were considered potential users of SB and were directed to a product appropriateness question: “Which group of products would you be willing to try if they contained seafood byproducts?” Using the CATA method, consumers selected all applicable groups from the list of 12 product categories [21]: dairy products; bakery products; meat and meat products including poultry and game; fat and oil, and fat products; drink and beverages; snack and energy/protein bars; candy and confectionery, cereals and cereal products, seasoning mix, sauces and dressings; soup and gravy products (canned and frozen); fish and fish products including mollusks and crustaceans; pasta and noodles.

### 2.5. Statistical Analysis

Willingness-to-try responses were reported as percentage [of the total population sample] “yes” responses to GWTT, GWTTS, and GWTTSH questions, and as percentage [of potential SB consumers only] selection for each product category in the CATA list. Effects of informational cues were evaluated by marginal homogeneity tests. The Stuart-Maxwell test was first performed to demonstrate significant overall shifts in responses (yes/maybe/no) from one informed condition to the next, and the McNemar’s test evaluated increases in “yes” responses from GWTT to GWTTS, and from GWTTS to GWTTSH. Demographic data were reported as percentages of the total population sample and were used as predictors (regressors) of the GWTT, GWTTS, and GWTTSH responses in separate logistic regression models. Likewise, consumers’ emotional profiles were used as predictors of the GWTTS, GWTTS, and GWTTSH responses in separate logistic regression models, with each emotion treated as a numerical variable (from 1-9 on the 9-point scale, or 0 for “not applicable”). Therefore, two logistic regression models were fitted: one with the full set of demographics, and another with the full set of emotions as predictors. The variable inflation factor (VIF) diagnostic did not show any evidence of multicollinearity which could have skewed effect estimates. Relative appropriateness of each product category for incorporation of SB was determined by frequency of selection (% willingness-to-try) from the CATA list. Perceived risks were also reported as percentage [of reluctant/averse consumers] selection. Data were analyzed by Microsoft^®^ Excel^®^ (Version 2206 Build 16.0.15330.20260; Microsoft Corporation, Redmond, WA, USA) and SAS (Copyright© 2016 SAS Institute Inc., Cary, NC, USA). A significance level of α = 0.05 was used throughout.

## 3. Results and Discussion

### 3.1. Effects of Informational Cues on Willingness-to-Try Seafood Byproducts

**Figure 2:**
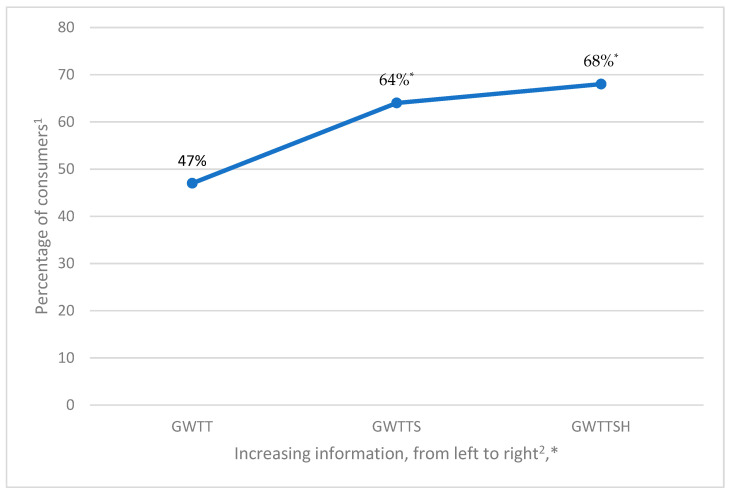
Willingness-to-try food containing seafood byproduct under different informed conditions. ^1^ Percentage of 904 consumer survey respondents. ^2^ GWTT: General willingness-to-try [with seafood byproduct definition only], GWTTS: General willingness-to-try [with definition and safety information], GWTTSH: General willingness-to-try [with definition, safety information, and health benefit information]. * There was a significant increase in “yes” responses (McNemar’s test) with additional information from GWTT to GWTTS (*p* < 0.001) and from GWTTS to GWTTSH (*p* < 0.01).

Initially, slightly less than half (47%) of the respondents (N = 904) reported “yes” for GWTT for food made with SB (Figure 2). Responses to the GWTT question were provided after presentation of a SB definition only, and it was hypothesized that additional SB informational cues could increase willingness-to-try.

With additional informational cue “…knowing it is safe for consumption,” the percentage of consumers willing to try food made with SB (GWTTS) increased from 47% to 64% (Figure 2). Concerns about the safety of novel foods (especially animal products) are documented reasons for avoidance, and for consumption to occur, assurances of safety precede sensory appeal [22]. Effectiveness of food safety information relies on credibility of the source and consumers’ experiences [23], and results (Figure 2) indicated that a significant proportion of respondents gave credence to the SB safety claim (Stuart Maxwell test, *p* < 0.001; McNemar’s test, *p* < 0.001). For these consumers, safety of SB was likely unknown, uncertain, or not salient before the given informational cue. This study demonstrated that consumers’ knowledge and trust in SB safety may be an important factor in product adoption (see later discussion of perceived safety risk).

Consumers’ positive GWTTSH significantly rose to 68% (Stuart Maxwell test, *p* < 0.001; McNemar’s test, *p* = 0.007), with the additional informational cue “…claimed to provide health benefits” (Figure 2). Other authors [24] found that information about public health increased openness to SB, whereas in this study the message was intended to appeal to individual health benefits. Various nutrients and health-promoting compounds have been identified in SB (see [10] for a review), and health-benefit claims have shown to be effective in increasing purchase intent for specific foods containing SB [17,25]. However, the magnitude of the effect may depend on overall product acceptability [25]. Since no actual food stimuli were presented in this research, the observed effects demonstrated attitudinal shifts in favor of SB consumption.

For this research, informational cues were defined as extrinsic messaging [23] which prompted decision-making based on limited information processing [26]. The observed positive impact of SB information cues (a safety claim for GWTTS, and a health benefit claim for GWTTSH) on SB trial intent suggested that they triggered interest and attention [21], were considered relevant and credible [23], and thus evoked more favorable judgements of SB among a significant proportion of consumers (Figure 2). When consumers are unfamiliar with a product, they often seek more information [16]. However, as the risk perception data will show, extrinsic information alone was not sufficient to overcome all the cognitive barriers to SB consumption.

### 3.2. Individual Predictors of Willingness-to-Try Seafood Byproducts

#### 3.2.1. Demographics

Based on logistic regression modeling, gender was found to be a consistently significant demographic predictor of general willingness-to-try responses across information levels (GWTT, GWTTS, and GWTTSH; Table 2). In each case, males were more likely to exhibit SB trial intent than females. However, the discrepancy between genders lessened with each additional informational cue, indicated by decreasing odds ratio (OR) estimates from 2.4 (GWTT) to 1.6 (GWTTS) to 1.4 (GWTTSH). While SB information increased “yes” responses for both genders, data suggested that it was more effective in changing the minds of female consumers. These findings supported suppositions that males are more open to novel foods, while females are more health and food-safety conscious [12].

For the purposes of this study, self-reported race was meant to reflect consumers’ social, cultural, and/or ethnic identity, rather than biological or genetic information [27]. All race categories exhibited a significant increase in willingness-to-try SB after informational cues had been given to them, yet the race variable revealed significant differences in frequency of GWTTS and GWTTSH responses (Table 2). As race was not a significant predictor of the initial GWTT response, its significance in subsequent models indicated that race was associated with differential responsiveness to informational cues regarding SB consumption.

Asian consumers exhibited the highest SB trial intent at each information level (62% GWTT, 76% GWTTS, and 77% GWTTSH), and Black/African American consumers reported the lowest (22% GWTT, 37% GWTTS, and 41% GWTTSH) despite being more responsive to informational cues (higher % increase in “yes” responses). As such, odds ratio (OR) estimates between Asian and Black/African American consumers decreased with increasing information, from GWTTS to GWTTSH (Table 2). It is possible that raw differences in WTT frequencies were due to relative novelty (vs. familiarity) of the food source. In Asian countries, seafood byproducts and coproducts have been more commonly available for human consumption than in the west [28]. In this study, US consumer status was not a significant predictor of WTT responses (Table 2), but it was worth noting that 85% of Black/African American respondents were US consumers, and 81% of Asian respondents were not.

Based on percent increase in “yes” responses to willingness-to-try questions, Hispanic/Latin/Spanish origin consumers were most positively responsive to informational cues regarding SB, increasing from 49% GWTT to 74% GWTTSH. After safety and health benefit claims, respectively, odds of GWTTS (OR = 1.6) and GWTTSH (OR = 1.9) were significantly higher for Hispanic/Latin/Spanish origin consumers than White consumers. Compared to Black/African American consumers, estimated odds of a “yes” response were more than twice as high for White (OR = 2.4 for GWTTS) and up to four times higher for Hispanic/Latin/Spanish origin consumers (OR=4.1 for GWTTSH; Table 2) during the period of this study. In previous research [17], combined messages about safety and health benefits of catfish byproduct had a greater impact on Hispanic/Latin Americans than US Americans. In the present study, self-reported race added resolution to statistical models, and evidenced that racial identity (or associated factors) can play a part in adoption of new foods such as SB, along with other individual factors.

Both reported knowledge of SB and previous SB consumption were associated with increased odds of trial intent across all informed conditions (Table 1). These effects were independent of race and gender (no significant interaction effects during model selection). It has been theorized that consumers’ level of familiarity with a product moderates their responsiveness to extrinsic cues [24]. In the present study, existing knowledge about SB had a cumulatively positive effect on trial intent along with informational cues, with OR estimates increasing from 1.6 (GWTT) to 1.7 (GWTTS) to 2.0 (GWTTSH; Table 2).

High OR estimates for previous SB consumers (OR ranging from 3.2-4.9) added to existing evidence that trial of a novel food can encourage future instances [13]. Positive impacts of SB knowledge and previous consumption further motivated the need for appropriate products containing SB which encourage initial trial. Through consumption behaviors, familiarity with SB can be increased, providing consumers with more complex schemas to evaluate actual product quality [29] and guide further development.

#### 3.2.2. Emotions

Food-related emotions can be studied from the perspective of how emotions affect food choice, or from the perspective of how food choice affects emotions [30]. This research was concerned with the former, using consumers’ emotional baseline scores (Table S1) during an early stage of the COVID-19 pandemic in 2020 as predictors of willingness-to-try foods containing SB. The COVID-19 outbreak impacted consumers’ emotions and food-related behaviors [31]. These included purchase and consumption habits [31] as well as food waste behavior [32]. The timing of similar online studies was considered a limitation to determine consumer perception and behavioral partners [15], but the current research was interested in how these unique conditions affected perceptions of waste-to-value foods made with SB. Still, a follow-up post COVID-19 pandemic online study would be warranted to investigate how emotional predictors of SB consumptions have changed, if at all.

Initially, only the emotion *bored* was significant in modeling the GWTT response (Table 3; full models including non-significant emotions are presented in supplementary Tables S2–S4). Strict interpretation of this relationship would imply that for every one-unit increase in *bored* (on a 9-point scale), estimated odds of GWTT = “yes” would increase by 10% (OR = 1.1). It was noticed that Asian consumers reported higher mean *bored* scores (6.7 on 9-point scale) during COVID-19 than each other group, while also reporting the highest GWTT intent. A study conducted in China in March, 2020 described associations between consumers’ boredom (due to limited activity during the COVID-19 “lockdown”), sensations seeking, and consumption willingness [33]. Thus, it is possible that increased boredom induced by the COVID-19 lockdown (Table S4) may have left consumers seeking new food experiences, and increased openness to SB consumption.

The baseline emotions, enthusiastic, free, and unsafe, became significant predictors of the GWTTS response, and free, loving, and unsafe were significant predictors of the GWTTSH response. Perhaps more relevant than the magnitude of these effects (all OR estimates between 0.89 and 1.1; Table 3) were their directions. In some instances, positive emotions would be expected to increase (expected OR > 1) and negative emotions expected to decrease (expected OR < 1) the motivation for consumption [30]. However, in the present case, feeling increasingly free or loving (positive emotions) was associated with reduced odds of willingness-to-try SB (OR < 1; Table 3), and feeling more unsafe (a negative emotion) was associated with higher willingness-to-try SB odds (OR > 1). These findings were interpreted in the context of pandemic-elicited emotions.

The significance of new terms after presentation of informational cues was indicative of the emotion profiles of consumers who were responsive to the safety and health claims. Consumers feeling more unsafe during the pandemic may have been reluctant to report positive GWTT and thus representative of those influenced by the safety claim. Grunert [23] posed that during non-crisis times, the food supply is considered safe, so safety has less influence on food choice. During a pandemic, however, many consumers took extra measures to ensure personal safety [31]. The SB safety information cue may have reassured some of these consumers, making unsafe a significant predictor of both GWTTS and GWTTSH responses. 

Similar to the *bored* emotion, activity restrictions imposed by the pandemic may have left consumers feeling less *free* and lacking in variety of experience [33]. These consumers with lower baseline *free* scores were seemingly influenced by informational cues, perhaps seeking new (but safe) food experiences. *Enthusiastic* has been called a sensation-seeking emotion [14] when presenting as a high-arousal motivator of new food experiences, and high states of arousal have been associated with attention to informational cues elsewhere [16]. Based on the GWTTS logistic regression model (Table 3), consumers feeling less *free* and/or more *enthusiastic* during the pandemic had higher odds of positive SB trial intent, perhaps related to the desire for variety and sensation under otherwise restricted conditions.

Objective explanations of lay persons’ self-reported emotions suffer from certain limitations [34]. First, there may not be consensus among scientists on definitions of emotional descriptors or terms. Perhaps more concerning is that naïve consumers with different linguistic backgrounds may use words and verbal scales differently; this can be exacerbated when translating from one language to another [34]. In the present internationally distributed online survey, it was unclear why the positive emotion *loving* became a significant predictor of the GWTTSH response with an OR estimate < 1, although Asian consumers reported significantly lower mean baseline *loving* scores along with the directionally highest proportion of GWTTSH = “yes” responses. Despite inherent limitations in psychological self-reporting, use of emotions to predict food choice does provide information beyond typical demographics and hedonic ratings [30]. In this study, openness to SB consumption and responsiveness to informational cues were affected by consumer emotions during the COVID-19 pandemic.

### 3.3. Product Appropriateness for Seafood Byproducts

**Figure 3:**
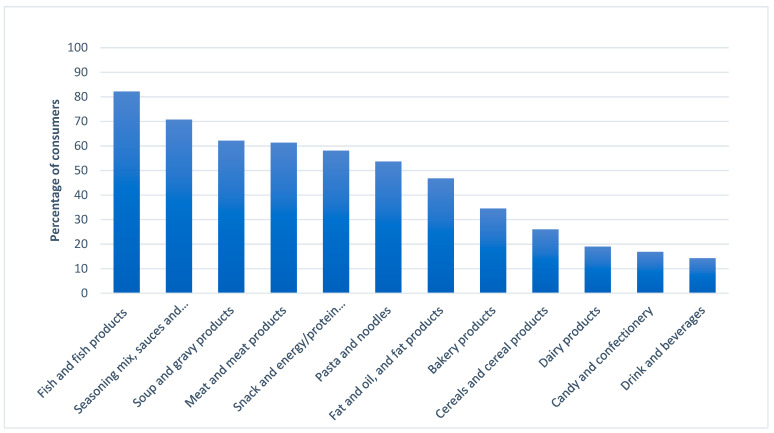
Product appropriateness of foods containing seafood byproduct based on willingness-to-try responses from 611 consumers.

Relative appropriateness of a product category was measured by frequency of selection from the CATA WTT list and reported as percentage of the 611 consumers who responded “yes” to the GWTTSH question. Among these potential SB consumers, 82% reported willingness-to-try *fish and fish products including mollusks, and crustaceans* if they contained SB, making it the most appropriate food product category among the twelve categories presented (Figure 3). After fish and fish products, the most appropriate categories were *seasoning mix, sauces and dressings* (71% WTT), *soup and gravy products (canned and frozen)* (62% WTT), *meat and meat products including poultry and game* (61% WTT), and *snack and energy/protein bars* (58% WTT). The median number of categories selected was 5, and the mode was 3, indicating that SB was considered appropriate for versatile applications by potential consumers.

Appropriateness has been called a situational aspect of acceptance and represents a cognitive dimension of food choice [35] separate from pure hedonic assessment. No actual products were evaluated in this study, so WTT responses were expected to be made based on mental constructs of hypothetical products, and appropriateness was intended to indicate a perceived fit of the ingredient (i.e., SB) within a final food product concept. In these situations, consumers may rely on expectations and/or past experiences when making their judgments [23].

For the sake of this discussion, the term seafood will be used to include finfish, crustaceans, mollusks, and other aquatic animals reared or captured for human consumption [3,4]. In this case, an edible seafood byproduct would still be inherently considered seafood, and potential consumers identified its most appropriate “fit” within its own product category (fish and fish products including mollusks, and crustaceans). In our previous research, catfish bone powder has found favorable acceptance when incorporated into breading mixes used to dredge fried catfish, and knowledge of its use increased purchase intent [9]. In another study [36], tilapia skins were made into ready-to-eat puffed snacks with only the addition of oil and seasonings, and in some parts of the world small fish bones have been eaten as snacks [3]. Within this product category, potential SB applications can include introduction into other seafood products [17], either overlapping with other categories (e.g., both seafood and snack) and/or standing alone as a new seafood product unto itself [36,37].

The seasoning mix, sauces, and dressings category was deemed second most appropriate for SB usage (Figure 3). Fish sauces, as an example, have been well-known and consumed in Asia and can be made using fish heads, bones, and entrails. Garum is thought to itself have originated as a byproduct of fish salting [37]. Beyond fermentation, various processing technologies have been used to transform would-be seafood waste into potential food ingredients, including oils which may lend to sauces/dressings, although sensory quality remains a consideration [10]. However, to determine the product appropriateness of SB, product developers should consider the market niche based on the consumer culture and demographic. Cross cultural studies with actual products containing SB should be conducted to provide clarity of the appropriateness of each category.

Among the top five product categories reported (Figure 3), examples of each can be found in literature (i.e., seafood, sauces, soups, sausage, snacks [3]). However, more widespread acceptance of foods made with SB will likely depend on a combination of perceived quality dimensions, such as appropriateness of usage (a search characteristic [11]), sensory quality (an experience characteristic [11]), and perceived product benefits (a credence characteristic [11]). Therefore, product development efforts should target the wants and needs of intended consumers, whether it be snacks fortified with powdered fish byproduct for school children in Ghana [38] or pâté made with fish roe, milt, and liver among teenagers in Ireland [39]. The present consumer-driven appropriateness data were intended as a starting point to guide effective SB valorization concepts which will encourage consumption.

### 3.4. Perceived Risks of Seafood Byproduct Consumption

When consumers make choices in the face of uncertainty (e.g., whether or not to try an unfamiliar food), they must judge the likelihood of negative consequences such as physical harm (safety), undesirable sensory experiences (taste, texture, etc.,), or negative social impacts and the severity of such outcomes, or perceived risks [16]. These subjective evaluations can lead consumers to avoid certain foods when perceived risk is high. Based on this theoretical approach to risk perception and consumer decision-making, the term risk has been used to describe barriers to novel food consumption [13].

**Figure 4:**
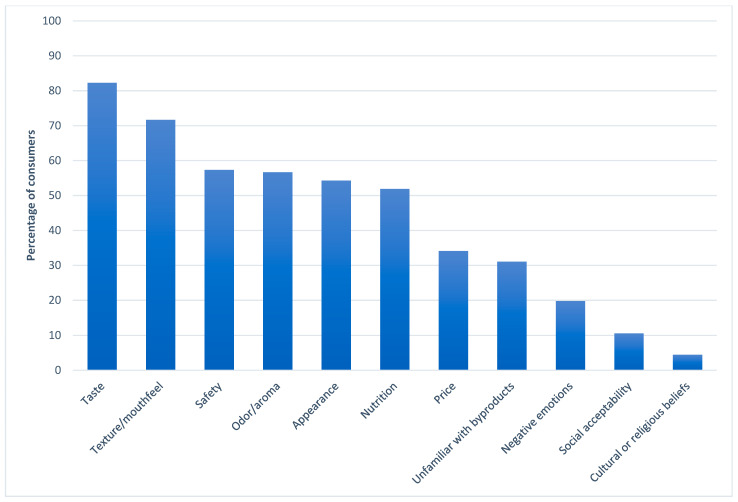
Perceived risks associated with seafood byproduct trial intent, based on responses from 293 consumers who were unwilling to try foods containing seafood byproducts.

Consumers who did not report willingness-to-try a SB-containing product after a safety claim and health benefit claim (“maybe” or “no” to the GWTTSH question) identified perceived risks, or reasons for their SB aversion from a CATA list of 11 items. Of these 293 consumers, 91% were considered SB-reluctant (GWTTSH = “maybe”) and 9% were considered SB-averse (GWTTSH = “no”). The frequency of risk selection was reported as percentages of 293 SB-reluctant/averse consumers (Figure 4). The median number of risks selected was 4, and the mode was 3.

Ninety-seven percent of reluctant/averse SB consumers cited at least one sensory attribute (taste, texture/mouthfeel, odor/aroma, or appearance) as a reason for SB avoidance. Taste is consistently a predominant driver of food choice, and was the most common reason for seafood byproduct avoidance (82% selection; Figure 4). Taste was followed by texture/mouthfeel (72% selection), odor/aroma (tied with safety at 57% selection), and appearance (54% selection; Figure 4) as the top cited SB risks. Lower sensory expectations of waste-to-value foods are not unique to SB, and have been shown for both plant [11] and animal [15] sourced byproducts. As only 60 of 293 SB-reluctant/averse consumers reported previous SB consumption, it was inferred that concerns about sensory quality were largely based on expectations rather than experience, and that SB was expected to be of inferior quality to more commonly consumed seafood portions. However, actual trials (Catfish nuggets/Fish skin snack) indicated that with the additional product information consumers accepted the product and these novel products have potential to be introduced to the market [14,36].

Despite an informational cue claiming that products containing SB are safe for consumption, safety was still the third most common reason for trial aversion (57% selection; Figure 4). Interestingly, consumers citing safety as a reason for SB avoidance reported lower unsafe emotion scores (a mean of 5.7 on a 9-point scale) than their counterparts who did not cite safety as a perceived risk (a mean unsafe score of 5.9). It was inferred that SB were considered less safe than typical seafood products by these consumers.

Food safety can be considered a credence characteristic, since the safety of a food is not directly experienced but must rely on trust [23]. As new technologies and uses for SB emerge, safety and risk management are priorities for industry [10,40] and nature of the material, production practices and processing methods, as well as appropriate testing [40] should be considered. Regulatory bodies also play a role in appropriate usage, labeling, and inspection of byproducts to ensure safety [41]. Labeling implications include identification of allergens [42] (i.e., fish or shellfish allergy) and any ingredient processing identification requirements (such as those existing for “mechanically separated” meat in the US) [41]. Reluctant/averse consumers’ trust in SB safety ultimately may depend on attitudes toward producers and regulations [23] as well as exposure, rather than simple claims. Concerns about nutrition of SB also remained an issue for 52% of reluctant/averse consumers, even after a health claim. As opposed to sensory quality (which is more subjective), content and recovery of nutrients and health-promoting compounds in SB has been empirically documented [10]. Fish and seafood have been considered healthy food choices [6], but based on the present data, SB has yet to be elevated to the same perceptions of healthfulness.

Remaining concerns of price, unfamiliarity, negative emotions, social acceptability, and cultural or religious beliefs were each cited less than 50% of the time, but are common barriers to acceptance of food from unusual sources. Some of these issues have been related to individual traits, such as food neophobia (aversion to new or unusual foods) [12,14], disgust sensitivity [14], or socio-cultural characteristics [15] elsewhere. While personal traits may be more stable, unfamiliarity can be overcome through exposure [14]. Additionally, education and proper communication regarding the SB benefits may help minimize misconceptions about risk related to SB consumption [13]. In all but three instances, each of these lesser cited risks was selected alongside more prevalent concerns of sensory quality, safety and/or nutrition of SB.

## 4. Concluding Remarks and Future Prospects

Not all, but most, seafood consumers participated in this study were willing to try foods made with SB. Much reluctance was overcome through simple informational cues, and potential consumers identified fish products as the best cognitive “fit” for seafood byproduct utilization. Initial openness to SB consumption may be best predicted by previous experience, while responsiveness to SB safety and health information was associated with individual factors (e.g., gender, race). Additionally, salient emotions can help predict seafood byproduct trial intent, which may have been motivated by a desire for varied experience during the COVID-19 pandemic. Barriers to SB trial intent can largely be overcome through cognitive strategies, but some consumers remain unconvinced of sensory appeal (mainly taste) and safety.

This experiment provided meaningful insight for product developers to create new products with SB guided by consumer-by-consumer data. However, results should not be generalized outside of the current population samples. Participants were unpaid volunteers, and the data were self-reported. The research was performed during the COVID-19 pandemic when participants may have faced many restrictions. No actual products were used to evaluate the WTT (GWTT, GWTTS, and GWTTSH), risks, or appropriateness in this study. Currently, seafood byproducts do not face any special labeling regulations other than reporting the ingredient and allergenicity. Agencies may work within each country to establish appropriate requirements to help the seafood industry commercialize new products containing SB. Future studies should be focused on studying consumer perception in actual trials with a specific food matrix for each byproduct.

## Figures and Tables

**Table 1:** Demographic and socioeconomic information of participants (N = 904).

Demographic and Socioeconomic Data		
Age (%)		
19-25 years	17.04	
26-35 years	36.50	
36-45 years	15.82	
46-55 years	15.49	
56-65 years	8.96	
>65 years	6.19	
Race (%)		
White	38.61	
Hispanic/Latin/Spanish origin	37.06	
Asian	19.47	
Black/African American	2.99	
Other	1.88	
Education Level (%)		
Graduate degree (MS, Ph.D., ED)	58.52	
College or equivalent	35.62	
High School diploma or below	5.86	
Gender (%)		
Female	62.94	
Male	37.06	
Employment status (%)	Before COVID-19	During COVID-19 *
Employed Full time, on-site	64.05	17.37
Employed Full-time, work from home	6.75	45.35
Employed Part-time on-site	8.74	3.32
Employed Part-time, work from home	3.98	10.4
Retired	7.19	7.3
Unemployed	9.29	16.26

* Data were collected online from April to June of 2020.

**Table 2:** Demographic predictors of willingness-to-try ^1^ food products containing seafood byproducts, based on N = 904 consumer responses.

	GWTT		GWTTS		GWTTSH	
Parameter	95% CI ^2^	OR ^3^	95% CI	OR	95% CI	OR
Gender (M:F)	1.74–3.22	2.4	1.18–2.20	1.6	1.047–1.99	1.4
US consumer	0.77–1.65	-	-	-	-	-
Race ^4^	-	-	-		-	
A:B	-	-	1.39–9.32	3.6	1.08–7.25	2.8
H:B	-	-	1.56–9.15	3.8	1.71–9.99	4.1
W:B	-	-	1.01–5.74	2.4	-	-
O:B	-	-	-	-	1.12–19.64	4.7
H:W	-	-	1.06–2.32	1.6	1.28–2.86	1.9
Previous ^5^	3.52–6.74	4.9	2.27–4.50	3.2	2.30–4.73	3.3
Knowledge ^6^	1.14–2.29	1.6	1.21–4.49	1.7	1.44–2.84	2.0

^1^ Based on separate logistic regression models of responses: GWTT = General willingness-to-try [with seafood byproduct definition only], GWTTS = General willingness-to-try [with definition and safety information], and GWTTSH = General willingness-to-try [with definition, safety information, and health benefit information]. ^2^ 95% Confidence intervals. ^3^ Odds ratio (OR; relative odds of a “yes” response) estimates were only presented and interpreted for predictors found to be significant to the model (*p*-value ≤ 0.05). ^4^ Significant between-group effects are presented for self-identified Asian:Black/African American (A:B), Hispanic/Latin/Spanish origin:Black/African American (H:B), White:Black/African American (W:B), Other race:Black/African American (O:B), and Hispanic/Latin/Spanish origin:White consumers (H:B). ^5^ Previous consumption of seafood byproducts (yes:no).^6^ Self-reported initial knowledge of seafood byproducts (yes:no).

**Table 3:** Significant emotional predictors ^1^ of willingness-to-try ^2^ foods containing seafood byproducts ^2^.

Response	Emotion	95% CI ^3^	Odds Ratio
GWTT	Bored	1.01–1.14	1.1
GWTTS	Enthusiastic	1.01–1.21	1.1
	Free	0.83–0.96	0.89
	Unsafe	1.00–1.16	1.1
GWTTSH	Free	0.84–0.92	0.88
	Loving	0.84–1.00	0.92
	Unsafe	1.04–1.03	1.1

^1^ Based on logistic regression models with 21 emotional predictors. Significance of terms was determined by Type III Wald tests (*p*-value ≤ 0.05). ^2^ GWTT = general willingness-to-try [with seafood byproduct definition only], GWTTS = general willingness-to-try [with definition and safety information], and GWTTSH = general willingness-to-try [with definition, safety information, and health benefit information]. ^3^ 95% Confidence intervals.

## Data Availability

The data that support the findings of this study are available from the corresponding author upon reasonable request.

## Supplementary Materials

The following supporting information can be downloaded at: https://www.mdpi.com/article/10.3390/foods12061313/s1. Table S1: Mean and standard deviation of emotions by race on a scale of 9-points. Table S2: Logistic regression model of general willingness-to-try (GWTT) foods containing seafood byproducts. Table S3: Logistic regression model of general willingness-to-try with safety claim (GWTTS) foods containing seafood byproducts. Table S4: Logistic regression model of general willingness-to-try with safety and health benefit claim (GWTTSH), foods containing seafood byproducts. All authors have read and agreed to the published version of the manuscript.
