# Supplementary material for: Factors Influencing Consumers’ Willingness-to-Try Seafood Byproducts
Source: Foods. 2023 Mar 20;12(6):1313. doi: 10.3390/foods12061313 (PMC10048574; doi:10.3390/foods12061313)
Supplement: Supplementary file 1 [file foods-12-01313-s001.zip › foods-2299867-supplementary.pdf]

Table S1. Mean and standard deviation of emotions by race on a scale of 9-points

Race/Emotions	White	Hispanic	Asian	Black/African American	Other	Overall
Active	5.51±2.47a	5.47±2.37a	4.49±2.11b	5.54±2.40ab	4.88±2.74ab	5.29±2.39
Adventurous[NS]	4.28±2.40	4.08±2.36	4.07±2.11	4.00±2.11	4.88±2.42	4.16±2.33
Aggressive	3.38±2.09b	3.37±2.13b	4.52±2.15a	4.29±2.18ab	2.50±1.87b	3.61±2.17
Bored	5.46±2.54b	5.62±2.47b	6.66±1.97a	5.69±2.60ab	4.88±2.83b	5.77±2.44
Calm	5.52±2.06a	5.30±2.15ab	4.82±2.11b	5.78±1.91ab	5.18±2.74ab	5.29±2.12
Eager	5.40±2.14a	5.14±2.21a	4.46±1.96b	5.40±2.18ab	5.81±2.14ab	5.15±2.15
Energetic	5.04±2.17a	5.15±2.24a	4.27±2.01b	4.73±2.11ab	4.65±2.87ab	4.93±2.19
Enthusiastic	4.79±2.02ab	5.06±2.19a	4.44±2.00b	4.76±2.22ab	4.81±3.17ab	4.83±2.09
Free[NS]	3.91±2.32	3.76±2.36	3.87±2.34	4.92±2.10	5.06±2.98	3.85±2.33
Friendly	6.36±1.89a	6.05±2.03a	4.90±2.15b	6.69±1.35a	5.82±2.79ab	5.95±2.07
Glad	4.81±2.11a	5.20±2.30a	3.67±2.06b	5.52±1.90a	5.76±2.44a	4.74±2.25
Good	5.97±1.82a	5.84±2.04a	4.53±2.09b	6.27±1.25a	6.24±2.44a	5.63±2.04
Healthy	6.25±2.05a	6.15±208a	4.64±2.13b	6.62±1.63a	6.18±2.60a	5.88±2.17
Happy	5.62±1.96a	5.44±2.11a	4.36±2.06b	6.00±1.88a	5.94±2.28a	5.29±2.10
Loving	6.61±1.81a	6.22±2.06a	5.05±2.22b	6.46±1.98a	5.76±2.61ab	6.14±2.08
Nostalgic	5.96±2.09ab	6.32±2.24a	5.57±2.16b	6.16±1.82ab	6.18±1.98ab	6.02±2.18
Peaceful	5.29±2.0ab	5.29±2.3ab	4.77±2.3b	6.04±1.64a	5.76±2.59ab	5.19±2.19
Pleased	4.66±2.05a	4.80±2.27a	4.10±2.13b	5.36±1.73a	5.41±2.53ab	4.60±2.17
Satisfied	4.97±2.05a	4.89±2.17a	3.88±2.10b	5.19±1.83a	4.94±3.00ab	4.72±2.15
Unsafe	4.69±2.38c	5.45±2.28b	6.25±1.93a	5.15±2.13abc	4.27±2.71abc	5.30±2.33
Worried	6.00±2.14ab	6.21±2.25b	6.64±1.80a	5.65±1.90ab	5.76±2.70ab	6.21±2.13

NS No significant difference in Race (White, Hispanic/Latin/Spanish origin, Asian), p-value <0.05
abc Difference in Race (White, Hispanic/Latin/Spanish origin, Asian), p-value <0.05

Table S2. Logistic regression model of general willingness to try (GWTT) foods containing seafood byproducts

Parameter	Estimate	P-value[1]	Odds ratio
Active	0.0078	0.84	1.0
Adventurous	0.035	0.31	1.0
Aggressive	-0.045	0.16	0.96
Bored	0.063	0.033	1.1
Calm	0.040	0.37	1.0
Eager	-0.028	0.41	0.97
Energetic	-0.024	0.61	0.98
Enthusiastic	0.070	0.12	1.1
Free	-0.022	0.53	0.98
Friendly	-0.017	0.71	0.98
Glad	0.0008	0.98	1.0
Good	0.019	0.72	1.0
Healthy	-0.013	0.76	0.99
Happy	-0.082	0.13	0.92
Loving	-0.021	0.59	0.98
Nostalgic	-0.033	0.28	0.97
Peaceful	-0.056	0.20	0.95
Pleased	0.0052	0.91	1.0
Satisfied	0.036	0.41	1.0
Unsafe	0.035	0.31	1.0
Worried	-0.062	0.093	0.94

[1] P-values based on Type III Wald tests

Table S3. Logistic regression model of general willingness to try, with safety claim (GWTTS), foods containing seafood byproducts

Parameter	Estimate	P-value[1]	Odds ratio
Active	-0.0085	0.84	0.99
Adventurous	0.0078	0.83	1.0
Aggressive	-0.024	0.48	0.98
Bored	0.023	0.45	1.0
Calm	0.048	0.31	1.0
Eager	0.013	0.71	1.0
Energetic	-0.070	0.16	0.93
Enthusiastic	0.098	0.04	1.1
Free	-0.12	0.0021	0.89
Friendly	-0.033	0.48	0.97
Glad	-0.012	0.79	0.99
Good	0.057	0.30	1.1
Healthy	-0.027	0.54	0.97
Happy	0.0024	0.97	1.0
Loving	-0.055	0.19	0.95
Nostalgic	0.0058	0.86	1.0
Peaceful	0.053	0.25	1.1
Pleased	-0.047	0.31	0.95
Satisfied	0.056	0.22	1.1
Unsafe	0.078	0.029	1.08
Worried	-0.031	0.42	0.97

[1] P-values based on Type III Wald tests

Table S4. Logistic regression model of general willingness to try, with safety and health benefit claim (GWTTSH), foods containing seafood byproducts

Parameter	Estimate	P-value[1]	Odds ratio
Active	-0.049	0.26	0.95
Adventurous	0.036	0.33	1.0
Aggressive	-0.012	0.72	0.99
Bored	0.019	0.53	1.0
Calm	0.059	0.22	1.1
Eager	0.026	0.49	1.0
Energetic	-0.029	0.58	0.97
Enthusiastic	0.062	0.21	1.1
Free	-0.10	0.0076	0.90
Friendly	0.012	0.80	1.0
Glad	0.067	0.14	1.1
Good	0.059	0.29	1.1
Healthy	-0.033	0.47	0.97
Happy	0.0065	0.91	1.0
Loving	-0.088	0.042	0.92
Nostalgic	0.034	0.30	1.0
Peaceful	0.020	0.68	1.0
Pleased	-0.067	0.16	0.94
Satisfied	-0.013	0.78	0.99
Unsafe	0.12	0.0016	1.1
Worried	-0.046	0.24	0.96

[1] P-values based on Type III Wald tests
